# Ethyl (3*E*)-3-[2-(4-bromo­phenyl­sulfon­yl)hydrazin-1-yl­idene]butano­ate

**DOI:** 10.1107/S1600536812019265

**Published:** 2012-05-05

**Authors:** Shahzad Murtaza, Naghmana Kausar, Aadil Abbas, M. Nawaz Tahir, Muhammad Zulfiqar

**Affiliations:** a University of Gujrat, Department of Chemistry, Hafiz Hayat Campus, Gujrat, Pakistan; b University of Sargodha, Department of Physics, Sargodha, Pakistan

## Abstract

The asymmetric unit of title compound, C_12_H_15_BrN_2_O_4_S, contains two mol­ecules (*A* and *B*), with slightly different conformations: the bromo­phenyl rings and the SO_2_ planes of the sulfonyl groups are oriented at dihedral angles of 50.2 (2) (mol­ecule *A*) and 58.24 (7)° (mol­ecule *B*), and the ethyl acetate groups make dihedral angles of 63.99 (19)° (*A*) and 65.35 (16)° (*B*) with their bromo­phenyl groups. In the crystal, both mol­ecules exist as inversion dimers linked by pairs of N—H⋯O hydrogen bonds, which generate *R*_2_^2^(14) loops. The dimers are linked by C—H⋯O inter­actions.

## Related literature

For a related structure, see: Uramoto *et al.* (1971 ▶). For hydrogen-bond motifs, see: Bernstein *et al.* (1995 ▶).
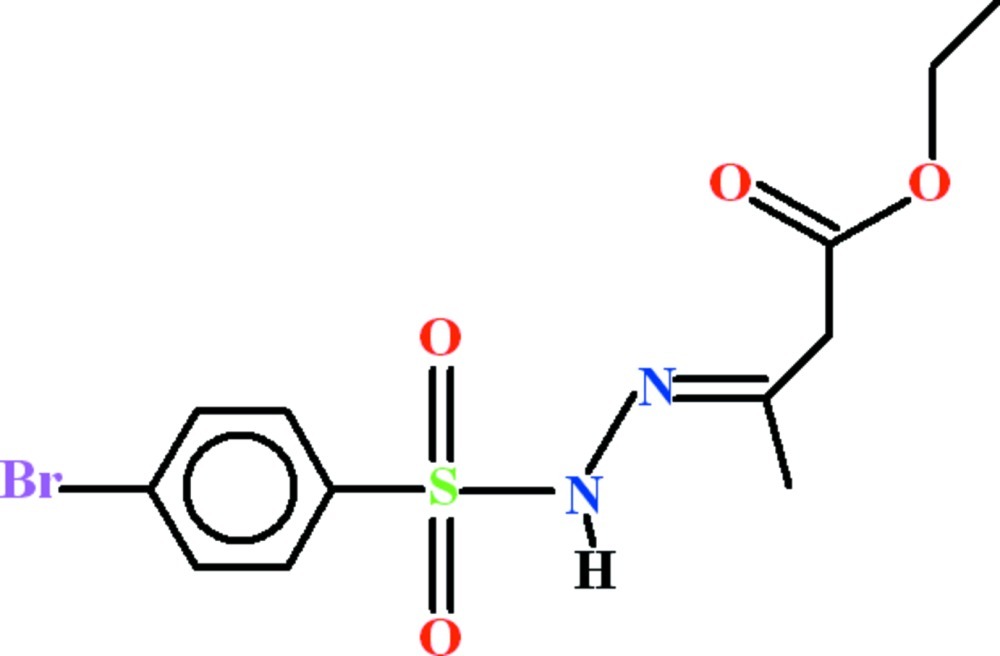


## Experimental

### 

#### Crystal data

C_12_H_15_BrN_2_O_4_S
*M* *_r_* = 363.23
Triclinic, 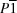

*a* = 11.0550 (3) Å
*b* = 11.9763 (4) Å
*c* = 13.1146 (3) Å
α = 77.457 (1)°
β = 72.163 (2)°
γ = 73.981 (1)°
*V* = 1571.92 (8) Å^3^
*Z* = 4
Mo *K*α radiation
μ = 2.76 mm^−1^
*T* = 296 K
0.30 × 0.15 × 0.14 mm

#### Data collection

Bruker Kappa APEXII CCD diffractometer
Absorption correction: multi-scan (*SADABS*; Bruker, 2005 ▶) *T*_min_ = 0.635, *T*_max_ = 0.650
22120 measured reflections
6096 independent reflections
4293 reflections with *I* > 2σ(*I*)
*R*_int_ = 0.030

#### Refinement

*R*[*F* ^2^ > 2σ(*F* ^2^)] = 0.049
*wR*(*F* ^2^) = 0.135
*S* = 1.05
6096 reflections
344 parameters
H-atom parameters constrained
Δρ_max_ = 1.27 e Å^−3^
Δρ_min_ = −1.11 e Å^−3^

### 

Data collection: *APEX2* (Bruker, 2009 ▶); cell refinement: *SAINT* (Bruker, 2009 ▶); data reduction: *SAINT*; program(s) used to solve structure: *SHELXS97* (Sheldrick, 2008 ▶); program(s) used to refine structure: *SHELXL97* (Sheldrick, 2008 ▶); molecular graphics: *ORTEP-3 for Windows* (Farrugia, 1997 ▶) and *PLATON* (Spek, 2009 ▶); software used to prepare material for publication: *WinGX* (Farrugia, 1999 ▶) and *PLATON*.

## Supplementary Material

Crystal structure: contains datablock(s) global, I. DOI: 10.1107/S1600536812019265/hb6771sup1.cif

Structure factors: contains datablock(s) I. DOI: 10.1107/S1600536812019265/hb6771Isup2.hkl

Supplementary material file. DOI: 10.1107/S1600536812019265/hb6771Isup3.cml

Additional supplementary materials:  crystallographic information; 3D view; checkCIF report

## Figures and Tables

**Table 1:** Hydrogen-bond geometry (Å, °)

*D*—H⋯*A*	*D*—H	H⋯*A*	*D*⋯*A*	*D*—H⋯*A*
N1—H1⋯O3^i^	0.86	2.46	2.909 (5)	114
N3—H3*A*⋯O7^ii^	0.86	2.36	2.8855 (13)	120
C3—H3⋯O4^iii^	0.93	2.53	3.450 (6)	172

Symmetry codes: (i) 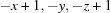; (ii) 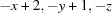; (iii) 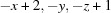.

**Fig. 1.**
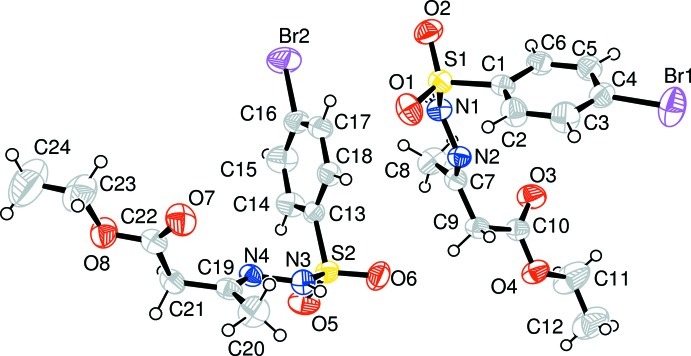
View of the title compound with displacement ellipsoids drawn at the 50% probability level.

**Fig. 2.**
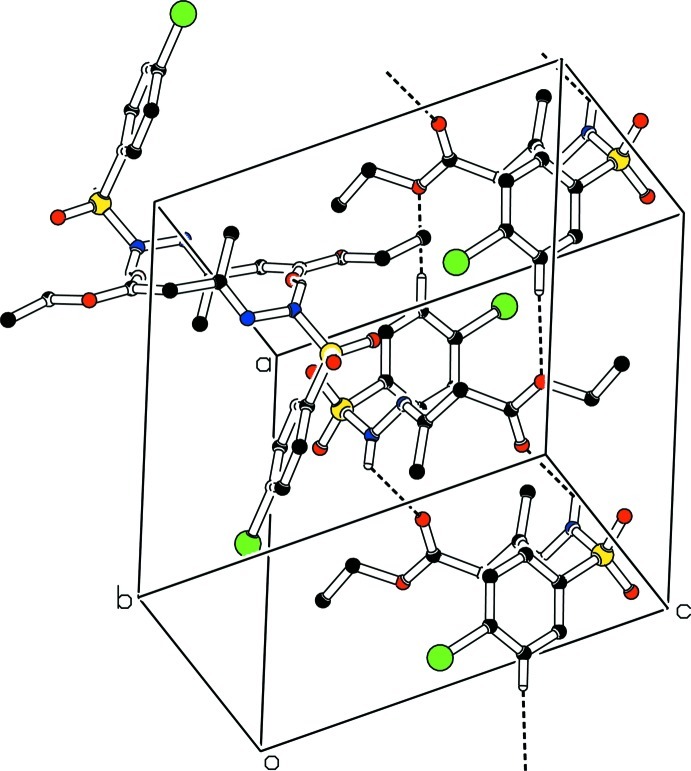
The partial packing, which shows that molecules are dimersed and then polymeric chains are formed. The H-atoms not involved in H-bondings are omitted for clarity.

## supplementary crystallographic information

### Comment

The title compound, (I) (Fig. 1), has been synthesized for the study of enzyme inhibation and other biological activities owing to the concept that sulfonyl hydrazide moiety is an important part of different drugs.

The crystal structure of bundlin A *p*-bromophenylsulfonylhydrazone (Uramoto *et al.*, 1971) has been published which contains the common moiety 4-bromo-*N*'-(propan-2-ylidene)benzenesulfonohydrazide which is related to the title compound (I, Fig. 1).

In (I), two molecules in the asymmetric unit are present, which differ from each other geometrically. In one molecule, the moieties of brompophenyl A (C1–C6/BR1), ethylidenehydrazine B (N1/N2/C7/C8) and ethyl acetate C (C9/C10/O3/O4/C11/C12) are almost planar with r. m. s. deviation of 0.0255, 0.0078 and 0.0398 Å, respectively. The dihedral angle between A/B, A/C and B/C is 86.14 (13)°, 54.81 (14)° and 65.35 (16)°, respectively. The sulfonyl group D (O1/S1/O2) is of course planar and makes a dihydral angle of 50.23 (21)° with its parent brompophenyl moiety. In second molecule, the similar moieties E (C13–C18/BR2), F (N3/N4/C19/C20) and G (C21/C22/O7/O8/C23/C24) are planar with r. m. s. deviation of 0.0059, 0.0016 and 0.0522 Å, respectively. The dihedral angle between E/F, E/G and F/G is 88.25 (10)°, 58.35 (14)° and 63.99 (19)°, respectively. The sulfonyl group H (O5/S2/O6) makes a dihydral angle of 58.24 (7)° with its parent brompophenyl E moiety. Both molecules are dimerized with themselves due to classical H-bondings of N—H···O type with *R*_2_^2^(14) (Table 1, Fig. 2) ring motifs (Bernstein *et al.*, 1995). The dimers of molecules containing bromine atom BR1 are interlinked due to C—H···O type of H-bondings, where CH is of phenyl ring and O-atom is of ester group.

### Experimental

A mixture of 4-bromobenzenesulphonohydrazide (0.251 g, 1 mmol) and ethyl acetoacetate (0.130 g, 1 mmol) in ethanol (20 ml) was refluxed for 4 h and then cooled to room temperature. After cooling, the solvent was removed under reduced pressure and the colorless solid residue was obtained. The resulting product was re-crystallized in ethanol and obtained colorless prisms of (I). m.p. 388 K.

### Refinement

The H-atoms were positioned geometrically (N—H = 0.86, C–H = 0.93–0.97 Å) and were included in the refinement in the riding model approximation, with *U*_iso_(H) = *xU*_eq_(C, N), where *x* = 1.5 for CH_3_ and *x* = 1.2 for other H-atoms.

### Crystal data

**Table:**

C_12_H_15_BrN_2_O_4_S	*Z* = 4
*M**_r_* = 363.23	*F*(000) = 736
Triclinic, *P*1	*D*_x_ = 1.535 Mg m^−^^3^
Hall symbol: -P 1	Mo *K*α radiation, λ = 0.71073 Å
*a* = 11.0550 (3) Å	Cell parameters from 4293 reflections
*b* = 11.9763 (4) Å	θ = 2.0–26.0°
*c* = 13.1146 (3) Å	µ = 2.76 mm^−^^1^
α = 77.457 (1)°	*T* = 296 K
β = 72.163 (2)°	Prism, colourless
γ = 73.981 (1)°	0.30 × 0.15 × 0.14 mm
*V* = 1571.92 (8) Å^3^	

### Data collection

**Table:**

Bruker Kappa APEXII CCD diffractometer	6096 independent reflections
Radiation source: fine-focus sealed tube	4293 reflections with *I* > 2σ(*I*)
Graphite monochromator	*R*_int_ = 0.030
Detector resolution: 7.80 pixels mm^-1^	θ_max_ = 26.0°, θ_min_ = 2.0°
ω scans	*h* = −13→13
Absorption correction: multi-scan (*SADABS*; Bruker, 2005)	*k* = −14→14
*T*_min_ = 0.635, *T*_max_ = 0.650	*l* = −16→16
22120 measured reflections	

### Refinement

**Table:**

Refinement on *F*^2^	Primary atom site location: structure-invariant direct methods
Least-squares matrix: full	Secondary atom site location: difference Fourier map
*R*[*F*^2^ > 2σ(*F*^2^)] = 0.049	Hydrogen site location: inferred from neighbouring sites
*wR*(*F*^2^) = 0.135	H-atom parameters constrained
*S* = 1.05	*w* = 1/[σ^2^(*F*_o_^2^) + (0.0612*P*)^2^ + 1.5738*P*] where *P* = (*F*_o_^2^ + 2*F*_c_^2^)/3
6096 reflections	(Δ/σ)_max_ = 0.001
344 parameters	Δρ_max_ = 1.27 e Å^−^^3^
0 restraints	Δρ_min_ = −1.11 e Å^−^^3^

### Special details

**Table:**

Geometry. Bond distances, angles *etc*. have been calculated using the rounded fractional coordinates. All su's are estimated from the variances of the (full) variance-covariance matrix. The cell e.s.d.'s are taken into account in the estimation of distances, angles and torsion angles
Refinement. Refinement of *F*^2^ against ALL reflections. The weighted *R*-factor *wR* and goodness of fit *S* are based on *F*^2^, conventional *R*-factors *R* are based on *F*, with *F* set to zero for negative *F*^2^. The threshold expression of *F*^2^ > σ(*F*^2^) is used only for calculating *R*-factors(gt) *etc*. and is not relevant to the choice of reflections for refinement. *R*-factors based on *F*^2^ are statistically about twice as large as those based on *F*, and *R*- factors based on ALL data will be even larger.

### Fractional atomic coordinates and isotropic or equivalent isotropic displacement parameters (Å^2^)

**Table:**

	*x*	*y*	*z*	*U*_iso_*/*U*_eq_	
Br1	1.11769 (5)	−0.38812 (5)	0.43815 (4)	0.0788 (2)	
S1	0.78563 (9)	0.03587 (8)	0.17990 (7)	0.0411 (3)	
O1	0.8641 (3)	0.1199 (2)	0.1313 (2)	0.0557 (10)	
O2	0.7263 (3)	−0.0035 (3)	0.1162 (2)	0.0558 (10)	
O3	0.5216 (3)	0.0584 (3)	0.6384 (2)	0.0629 (11)	
O4	0.6201 (3)	0.1478 (3)	0.7100 (2)	0.0532 (10)	
N1	0.6630 (3)	0.0928 (3)	0.2748 (2)	0.0397 (10)	
N2	0.7025 (3)	0.1166 (3)	0.3597 (2)	0.0384 (10)	
C1	0.8795 (3)	−0.0857 (3)	0.2460 (3)	0.0381 (11)	
C2	1.0045 (4)	−0.0849 (4)	0.2421 (4)	0.0544 (14)	
C3	1.0752 (4)	−0.1770 (4)	0.2979 (4)	0.0597 (16)	
C4	1.0203 (4)	−0.2686 (3)	0.3560 (3)	0.0461 (12)	
C5	0.8954 (4)	−0.2710 (4)	0.3593 (3)	0.0482 (12)	
C6	0.8250 (4)	−0.1790 (3)	0.3030 (3)	0.0453 (12)	
C7	0.6106 (3)	0.1691 (3)	0.4305 (3)	0.0396 (11)	
C8	0.4704 (4)	0.2101 (4)	0.4284 (3)	0.0586 (16)	
C9	0.6502 (4)	0.1910 (3)	0.5228 (3)	0.0424 (12)	
C10	0.5890 (3)	0.1252 (3)	0.6290 (3)	0.0419 (12)	
C11	0.5598 (6)	0.0940 (7)	0.8188 (4)	0.099 (3)	
C12	0.6102 (3)	0.1162 (2)	0.89744 (13)	0.109 (3)	
Br2	0.35796 (4)	0.36766 (4)	0.06489 (4)	0.0807 (2)	
S2	0.68585 (4)	0.53305 (5)	0.30636 (4)	0.0406 (3)	
O5	0.60925 (4)	0.63906 (5)	0.34696 (4)	0.0562 (10)	
O6	0.73411 (5)	0.43392 (7)	0.37752 (5)	0.0600 (10)	
O7	0.99883 (6)	0.64595 (9)	−0.14623 (7)	0.0629 (11)	
O8	0.89569 (5)	0.82270 (6)	−0.20911 (5)	0.0571 (10)	
N3	0.81726 (5)	0.56299 (7)	0.21809 (5)	0.0401 (10)	
N4	0.7935 (3)	0.6522 (3)	0.1321 (2)	0.0384 (9)	
C13	0.5960 (3)	0.4894 (3)	0.2375 (3)	0.0378 (11)	
C14	0.4690 (4)	0.5485 (4)	0.2418 (4)	0.0541 (16)	
C15	0.3982 (4)	0.5113 (4)	0.1912 (4)	0.0646 (16)	
C16	0.4555 (4)	0.4169 (4)	0.1368 (3)	0.0513 (14)	
C17	0.5823 (4)	0.3571 (3)	0.1316 (3)	0.0498 (14)	
C18	0.6528 (4)	0.3938 (3)	0.1828 (3)	0.0448 (12)	
C19	0.8945 (4)	0.6858 (3)	0.0688 (3)	0.0411 (12)	
C20	1.0308 (4)	0.6400 (4)	0.0792 (4)	0.0603 (16)	
C21	0.8693 (4)	0.7805 (3)	−0.0225 (3)	0.0442 (12)	
C22	0.9295 (4)	0.7404 (3)	−0.1316 (3)	0.0428 (12)	
C23	0.9492 (6)	0.7938 (5)	−0.3193 (4)	0.088 (2)	
C24	0.8877 (10)	0.8834 (7)	−0.3902 (5)	0.151 (4)	
H1	0.58287	0.10678	0.27334	0.0476*	
H2	1.04121	−0.02258	0.20198	0.0649*	
H3	1.15997	−0.17705	0.29617	0.0714*	
H5	0.85930	−0.33381	0.39886	0.0579*	
H6	0.74091	−0.17969	0.30335	0.0542*	
H8A	0.46525	0.24267	0.35579	0.0878*	
H8B	0.42710	0.26895	0.47500	0.0878*	
H8C	0.42897	0.14484	0.45306	0.0878*	
H9A	0.62468	0.27440	0.52727	0.0511*	
H9B	0.74440	0.16716	0.50890	0.0511*	
H11A	0.46620	0.12524	0.83593	0.1181*	
H11B	0.57574	0.00993	0.82031	0.1181*	
H12A	0.70237	0.08301	0.88193	0.1631*	
H12B	0.56803	0.08127	0.96785	0.1631*	
H12C	0.59457	0.19941	0.89594	0.1631*	
H3A	0.89452	0.52737	0.22352	0.0481*	
H14	0.43149	0.61308	0.27857	0.0650*	
H15	0.31208	0.54993	0.19406	0.0775*	
H17	0.61965	0.29300	0.09419	0.0597*	
H18	0.73855	0.35439	0.18051	0.0534*	
H20A	1.03026	0.62775	0.15418	0.0903*	
H20B	1.08237	0.69588	0.03942	0.0903*	
H20C	1.06744	0.56699	0.05082	0.0903*	
H21A	0.90367	0.84604	−0.02061	0.0530*	
H21B	0.77582	0.80815	−0.01255	0.0530*	
H23A	1.04258	0.78818	−0.34174	0.1054*	
H23B	0.93377	0.71873	−0.32190	0.1054*	
H24A	0.79493	0.89168	−0.36510	0.2268*	
H24B	0.91809	0.86258	−0.46188	0.2268*	
H24C	0.90870	0.95638	−0.39131	0.2268*	

### Atomic displacement parameters (Å^2^)

**Table:**

	*U*^11^	*U*^22^	*U*^33^	*U*^12^	*U*^13^	*U*^23^
Br1	0.0633 (3)	0.0840 (4)	0.0816 (4)	−0.0112 (3)	−0.0321 (3)	0.0156 (3)
S1	0.0424 (5)	0.0425 (5)	0.0351 (5)	−0.0108 (4)	−0.0027 (4)	−0.0082 (4)
O1	0.0520 (17)	0.0497 (17)	0.0538 (17)	−0.0190 (14)	0.0037 (13)	0.0009 (13)
O2	0.0610 (18)	0.0643 (19)	0.0442 (16)	−0.0064 (14)	−0.0166 (14)	−0.0179 (14)
O3	0.076 (2)	0.073 (2)	0.0516 (17)	−0.0494 (18)	−0.0108 (15)	−0.0003 (15)
O4	0.0545 (16)	0.075 (2)	0.0368 (14)	−0.0327 (15)	−0.0092 (12)	−0.0028 (13)
N1	0.0329 (16)	0.0461 (18)	0.0392 (17)	−0.0084 (14)	−0.0044 (13)	−0.0123 (14)
N2	0.0397 (17)	0.0412 (17)	0.0361 (16)	−0.0136 (14)	−0.0071 (14)	−0.0077 (14)
C1	0.039 (2)	0.039 (2)	0.0376 (19)	−0.0126 (16)	−0.0050 (16)	−0.0109 (16)
C2	0.046 (2)	0.052 (2)	0.067 (3)	−0.023 (2)	−0.015 (2)	0.003 (2)
C3	0.042 (2)	0.068 (3)	0.073 (3)	−0.024 (2)	−0.018 (2)	0.001 (2)
C4	0.044 (2)	0.051 (2)	0.045 (2)	−0.0073 (18)	−0.0155 (18)	−0.0092 (19)
C5	0.050 (2)	0.049 (2)	0.049 (2)	−0.0175 (19)	−0.0138 (19)	−0.0045 (19)
C6	0.039 (2)	0.049 (2)	0.053 (2)	−0.0160 (18)	−0.0125 (18)	−0.0092 (19)
C7	0.041 (2)	0.040 (2)	0.0349 (19)	−0.0133 (16)	−0.0021 (17)	−0.0056 (16)
C8	0.044 (2)	0.074 (3)	0.052 (3)	0.000 (2)	−0.008 (2)	−0.021 (2)
C9	0.045 (2)	0.046 (2)	0.038 (2)	−0.0197 (18)	−0.0028 (17)	−0.0095 (17)
C10	0.038 (2)	0.046 (2)	0.043 (2)	−0.0148 (18)	−0.0075 (17)	−0.0068 (18)
C11	0.107 (4)	0.164 (6)	0.040 (3)	−0.084 (4)	−0.012 (3)	0.012 (3)
C12	0.137 (6)	0.154 (6)	0.052 (3)	−0.068 (5)	−0.029 (4)	0.002 (4)
Br2	0.0752 (4)	0.0956 (4)	0.0968 (4)	−0.0393 (3)	−0.0383 (3)	−0.0181 (3)
S2	0.0412 (5)	0.0483 (6)	0.0339 (5)	−0.0158 (4)	−0.0065 (4)	−0.0066 (4)
O5	0.0492 (16)	0.0646 (19)	0.0545 (17)	−0.0135 (14)	0.0028 (14)	−0.0311 (15)
O6	0.0692 (19)	0.0684 (19)	0.0463 (16)	−0.0249 (16)	−0.0256 (14)	0.0111 (14)
O7	0.069 (2)	0.0466 (18)	0.0585 (18)	0.0060 (15)	−0.0043 (16)	−0.0199 (15)
O8	0.0664 (19)	0.0519 (17)	0.0427 (16)	−0.0043 (14)	−0.0066 (14)	−0.0082 (14)
N3	0.0303 (16)	0.0441 (18)	0.0438 (18)	−0.0091 (13)	−0.0073 (13)	−0.0044 (14)
N4	0.0411 (17)	0.0359 (16)	0.0374 (16)	−0.0107 (14)	−0.0044 (14)	−0.0095 (13)
C13	0.038 (2)	0.040 (2)	0.0370 (19)	−0.0138 (16)	−0.0103 (16)	−0.0019 (16)
C14	0.040 (2)	0.054 (3)	0.069 (3)	−0.0041 (19)	−0.012 (2)	−0.022 (2)
C15	0.038 (2)	0.076 (3)	0.084 (3)	−0.006 (2)	−0.021 (2)	−0.022 (3)
C16	0.051 (2)	0.059 (3)	0.054 (2)	−0.025 (2)	−0.020 (2)	−0.004 (2)
C17	0.056 (3)	0.043 (2)	0.055 (2)	−0.0112 (19)	−0.018 (2)	−0.0119 (19)
C18	0.041 (2)	0.042 (2)	0.051 (2)	−0.0057 (17)	−0.0147 (18)	−0.0069 (18)
C19	0.042 (2)	0.042 (2)	0.040 (2)	−0.0139 (17)	−0.0009 (17)	−0.0161 (17)
C20	0.039 (2)	0.078 (3)	0.063 (3)	−0.021 (2)	−0.008 (2)	−0.006 (2)
C21	0.049 (2)	0.038 (2)	0.041 (2)	−0.0146 (17)	0.0016 (18)	−0.0097 (17)
C22	0.039 (2)	0.042 (2)	0.045 (2)	−0.0122 (18)	−0.0008 (17)	−0.0120 (18)
C23	0.105 (4)	0.096 (4)	0.045 (3)	0.001 (3)	−0.009 (3)	−0.021 (3)
C24	0.223 (10)	0.125 (6)	0.077 (4)	0.040 (6)	−0.060 (6)	−0.027 (4)

### Geometric parameters (Å, º)

**Table:**

Br1—C4	1.887 (4)	C6—H6	0.9300
Br2—C16	1.901 (5)	C8—H8B	0.9600
S1—O2	1.424 (3)	C8—H8A	0.9600
S1—N1	1.636 (3)	C8—H8C	0.9600
S1—O1	1.427 (3)	C9—H9B	0.9700
S1—C1	1.766 (4)	C9—H9A	0.9700
S2—O6	1.4280 (9)	C11—H11B	0.9700
S2—N3	1.6297 (8)	C11—H11A	0.9700
S2—C13	1.765 (4)	C12—H12A	0.9600
S2—O5	1.4271 (8)	C12—H12B	0.9600
O3—C10	1.200 (5)	C12—H12C	0.9600
O4—C11	1.467 (6)	C13—C18	1.380 (5)
O4—C10	1.311 (5)	C13—C14	1.377 (6)
O7—C22	1.196 (4)	C14—C15	1.375 (7)
O8—C23	1.462 (5)	C15—C16	1.366 (6)
O8—C22	1.319 (4)	C16—C17	1.375 (6)
N1—N2	1.420 (4)	C17—C18	1.375 (6)
N2—C7	1.270 (5)	C19—C20	1.492 (7)
N1—H1	0.8600	C19—C21	1.496 (5)
N3—N4	1.409 (3)	C21—C22	1.503 (5)
N4—C19	1.278 (5)	C23—C24	1.425 (10)
N3—H3A	0.8600	C14—H14	0.9300
C1—C2	1.370 (6)	C15—H15	0.9300
C1—C6	1.380 (5)	C17—H17	0.9300
C2—C3	1.374 (7)	C18—H18	0.9300
C3—C4	1.369 (6)	C20—H20A	0.9600
C4—C5	1.376 (7)	C20—H20B	0.9600
C5—C6	1.375 (6)	C20—H20C	0.9600
C7—C9	1.501 (5)	C21—H21A	0.9700
C7—C8	1.498 (6)	C21—H21B	0.9700
C9—C10	1.503 (5)	C23—H23A	0.9700
C11—C12	1.413 (7)	C23—H23B	0.9700
C2—H2	0.9300	C24—H24A	0.9600
C3—H3	0.9300	C24—H24B	0.9600
C5—H5	0.9300	C24—H24C	0.9600
			
O1—S1—O2	120.04 (17)	O4—C11—H11B	109.00
O1—S1—N1	108.05 (17)	C12—C11—H11A	109.00
O1—S1—C1	107.54 (18)	H11A—C11—H11B	108.00
O2—S1—N1	104.25 (19)	O4—C11—H11A	109.00
O2—S1—C1	109.86 (19)	C12—C11—H11B	109.00
N1—S1—C1	106.29 (17)	C11—C12—H12B	109.00
O5—S2—O6	120.45 (5)	H12A—C12—H12C	109.00
O5—S2—N3	107.69 (5)	H12B—C12—H12C	109.00
O5—S2—C13	107.71 (12)	C11—C12—H12A	109.00
O6—S2—N3	103.84 (5)	C11—C12—H12C	110.00
O6—S2—C13	108.70 (12)	H12A—C12—H12B	109.00
N3—S2—C13	107.84 (13)	C14—C13—C18	120.7 (4)
C10—O4—C11	116.9 (4)	S2—C13—C14	119.8 (3)
C22—O8—C23	116.2 (3)	S2—C13—C18	119.4 (3)
S1—N1—N2	113.1 (3)	C13—C14—C15	119.4 (4)
N1—N2—C7	114.7 (3)	C14—C15—C16	119.4 (4)
S1—N1—H1	123.00	Br2—C16—C17	119.0 (3)
N2—N1—H1	124.00	C15—C16—C17	121.9 (4)
S2—N3—N4	114.28 (15)	Br2—C16—C15	119.2 (4)
N3—N4—C19	115.1 (3)	C16—C17—C18	118.7 (4)
S2—N3—H3A	123.00	C13—C18—C17	119.8 (4)
N4—N3—H3A	123.00	N4—C19—C20	126.0 (4)
C2—C1—C6	120.9 (4)	C20—C19—C21	118.9 (4)
S1—C1—C6	119.6 (3)	N4—C19—C21	115.1 (4)
S1—C1—C2	119.5 (3)	C19—C21—C22	113.0 (3)
C1—C2—C3	119.4 (4)	O7—C22—O8	124.3 (3)
C2—C3—C4	119.7 (4)	O7—C22—C21	124.4 (3)
C3—C4—C5	121.4 (4)	O8—C22—C21	111.3 (3)
Br1—C4—C5	120.7 (3)	O8—C23—C24	108.9 (5)
Br1—C4—C3	117.9 (4)	C13—C14—H14	120.00
C4—C5—C6	118.9 (4)	C15—C14—H14	120.00
C1—C6—C5	119.8 (4)	C14—C15—H15	120.00
C8—C7—C9	118.9 (3)	C16—C15—H15	120.00
N2—C7—C9	115.2 (3)	C16—C17—H17	121.00
N2—C7—C8	125.9 (3)	C18—C17—H17	121.00
C7—C9—C10	112.1 (3)	C13—C18—H18	120.00
O4—C10—C9	111.9 (3)	C17—C18—H18	120.00
O3—C10—C9	124.1 (3)	C19—C20—H20A	109.00
O3—C10—O4	124.0 (3)	C19—C20—H20B	109.00
O4—C11—C12	111.3 (5)	C19—C20—H20C	109.00
C3—C2—H2	120.00	H20A—C20—H20B	109.00
C1—C2—H2	120.00	H20A—C20—H20C	109.00
C4—C3—H3	120.00	H20B—C20—H20C	110.00
C2—C3—H3	120.00	C19—C21—H21A	109.00
C6—C5—H5	121.00	C19—C21—H21B	109.00
C4—C5—H5	121.00	C22—C21—H21A	109.00
C5—C6—H6	120.00	C22—C21—H21B	109.00
C1—C6—H6	120.00	H21A—C21—H21B	108.00
C7—C8—H8C	109.00	O8—C23—H23A	110.00
H8B—C8—H8C	109.00	O8—C23—H23B	110.00
H8A—C8—H8C	109.00	C24—C23—H23A	110.00
C7—C8—H8B	109.00	C24—C23—H23B	110.00
C7—C8—H8A	110.00	H23A—C23—H23B	108.00
H8A—C8—H8B	109.00	C23—C24—H24A	109.00
C7—C9—H9A	109.00	C23—C24—H24B	109.00
C10—C9—H9B	109.00	C23—C24—H24C	109.00
C10—C9—H9A	109.00	H24A—C24—H24B	110.00
C7—C9—H9B	109.00	H24A—C24—H24C	109.00
H9A—C9—H9B	108.00	H24B—C24—H24C	109.00
			
O1—S1—N1—N2	−63.0 (3)	N3—N4—C19—C21	180.0 (3)
O2—S1—N1—N2	168.3 (3)	S1—C1—C2—C3	176.9 (4)
C1—S1—N1—N2	52.2 (3)	C6—C1—C2—C3	−1.6 (7)
O1—S1—C1—C2	−0.5 (4)	S1—C1—C6—C5	−176.6 (3)
O1—S1—C1—C6	178.0 (3)	C2—C1—C6—C5	1.9 (6)
O2—S1—C1—C2	131.8 (3)	C1—C2—C3—C4	0.4 (7)
O2—S1—C1—C6	−49.8 (4)	C2—C3—C4—Br1	−176.2 (4)
N1—S1—C1—C2	−116.0 (4)	C2—C3—C4—C5	0.4 (7)
N1—S1—C1—C6	62.5 (3)	Br1—C4—C5—C6	176.5 (3)
C13—S2—N3—N4	59.0 (2)	C3—C4—C5—C6	−0.1 (6)
O5—S2—C13—C14	−7.2 (4)	C4—C5—C6—C1	−1.0 (6)
O5—S2—C13—C18	174.8 (3)	N2—C7—C9—C10	−115.5 (4)
O6—S2—C13—C14	124.8 (3)	C8—C7—C9—C10	64.9 (4)
O6—S2—C13—C18	−53.1 (3)	C7—C9—C10—O3	3.5 (5)
O5—S2—N3—N4	−57.04 (16)	C7—C9—C10—O4	−177.7 (3)
O6—S2—N3—N4	174.17 (15)	S2—C13—C14—C15	−177.7 (4)
N3—S2—C13—C18	58.9 (3)	C18—C13—C14—C15	0.2 (7)
N3—S2—C13—C14	−123.2 (3)	S2—C13—C18—C17	178.1 (3)
C11—O4—C10—C9	176.5 (4)	C14—C13—C18—C17	0.2 (6)
C11—O4—C10—O3	−4.8 (6)	C13—C14—C15—C16	−0.5 (7)
C10—O4—C11—C12	174.6 (4)	C14—C15—C16—Br2	−178.9 (4)
C23—O8—C22—O7	−0.5 (6)	C14—C15—C16—C17	0.4 (7)
C22—O8—C23—C24	171.0 (6)	Br2—C16—C17—C18	179.4 (3)
C23—O8—C22—C21	179.7 (4)	C15—C16—C17—C18	0.0 (6)
S1—N1—N2—C7	174.9 (3)	C16—C17—C18—C13	−0.3 (6)
N1—N2—C7—C8	−2.5 (5)	N4—C19—C21—C22	−113.9 (4)
N1—N2—C7—C9	177.9 (3)	C20—C19—C21—C22	66.6 (5)
S2—N3—N4—C19	172.6 (2)	C19—C21—C22—O7	−5.4 (6)
N3—N4—C19—C20	−0.5 (5)	C19—C21—C22—O8	174.4 (3)

### Hydrogen-bond geometry (Å, º)

**Table:**

*D*—H···*A*	*D*—H	H···*A*	*D*···*A*	*D*—H···*A*
N1—H1···O3^i^	0.86	2.46	2.909 (5)	114
N3—H3*A*···O7^ii^	0.86	2.36	2.8855 (13)	120
C3—H3···O4^iii^	0.93	2.53	3.450 (6)	172

Symmetry codes: (i) −*x*+1, −*y*, −*z*+1; (ii) −*x*+2, −*y*+1, −*z*; (iii) −*x*+2, −*y*, −*z*+1.
